# Mentorship, memory, and honor: Rita Levi-Montalcini’s legacy and the role of academic recognition in promoting women in science

**DOI:** 10.3389/fnmol.2026.1775988

**Published:** 2026-03-13

**Authors:** Léa Sun, Guila Delouya, Daniel Taussky

**Affiliations:** 1 John Abbott College, Sainte-Anne-de-Bellevue, QC, Canada; 2 Department of Radiation Oncology, Centre Hospitalier de l’Université de Montréal, Montréal, QC, Canada

**Keywords:** institutional honors, legacy, mentorship, Rita Levi-Montalcini, STEM

## Abstract

The contemporary vignette highlights how the mentorship model exemplified by Rita Levi-Montalcini continues to foster scientific identity, confidence, and career progression, especially among women and minorities facing systemic barriers. Through a mixed-methods narrative approach, combining historical-biographical review, qualitative analysis of a contemporary mentee vignette, and bibliometric insights. Levi-Montalcini’s own experience under Giuseppe Levi’s mentorship demonstrated the critical psychosocial and instrumental support necessary to develop resilience and scientific rigor. This research highlights how mentorship by Giuseppe Levi shaped Levi-Montalcini’s scientific rigor and resilience, contributing to her Nobel Prize-winning discoveries. Similarly, modern mentorship and institutional honors serve to counteract stereotype threats and enhance retention by providing recognition through awards and leadership roles, reflecting Levi-Montalcini’s legacy of transforming private perseverance into a public authority. This study underscores that structured mentorship programs and transparent recognition systems, inspired by Levi-Montalcini’s trajectory, are essential for universities to promote inclusive excellence and empower emerging women scientists in STEM (Science, Technology, Engineering, and Mathematics) fields.

## Introduction

Rita Levi-Montalcini’s remarkable journey from conducting clandestine research under fascist regime to becoming a Nobel laureate and honorary doctorate recipient exemplifies the transformative power of mentorship and symbolic recognition in science (Abikhzer et al., 2024). Rita Levi-Montalcini (RLM) left a lasting impact on many young researchers who held her in high regard (Aloe, 2021). This essay delves into how RLM was mentored by Professor Giuseppe Levi, who guided three future Nobel Prize winners, and explores Levi-Montalcini’s scientific development and resilience, highlighting how mentorship can shape not only careers but also one’s character. By analyzing Levi-Montalcini’s legacy as both a mentor and mentee and blending it with the personal experience of mentorship from a young aspiring scientist, this essay argues that mentorship and recognition are not merely personal milestones but institutional tools for fostering inclusive excellence, particularly for women in STEM (Casad et al., 2021). This study examines how scientific mentorship and symbolic recognition contribute to the advancement of women in STEM using Rita Levi-Montalcini’s example. The paper concludes with reflections on how universities can better support emerging scientists, especially women, through mentorship programs and meaningful honors. This study aims to explore how mentorship and symbolic academic recognition, as exemplified by Rita Levi-Montalcini’s trajectory from clandestine research to Nobel Laureate, function as mechanisms for advancing women in STEM fields. Through the example of Rita Levi-Montalcini and a modern mentee perspective, this study highlights how these mechanisms together foster inclusive excellence in science.

## Methods

This study used a mixed methods- narrative approach combining a structured historical and biographical review, a qualitative analysis of a contemporary mentee vignette, and an optional bibliometric comparison of mentee career outcomes. Below, we integrate the original methods with a clear description of how the Contemporary Mentee Perspective was collected, managed, and analyzed.

### Study design and overall approach

Design: Mixed methods- narrative synthesis linking historical case evidence with contemporary lived experience and, where feasible, quantitative bibliometric patterns.

Rationale: The combined approach was chosen to connect archival and published evidence about Rita Levi-Montalcini- and her mentor Giuseppe Levi with a first-person- vignette that illustrates the mechanisms of mentorship and institutional recognition in a modern context.

### Data sources and search strategy

Historical review: We searched PubMed, and Google Scholar for English and French sources through 2025 using keywords such as “Rita Levi-Montalcini,” “Giuseppe Levi,” “mentorship,” and “women in STEM.” The search was supplemented by targeted book searches.

Contemporary vignette: The vignette was provided directly by the study’s author.

### Inclusion and exclusion criteria

Historical materials: Included primary historical accounts, peer-reviewed- articles, authoritative reviews, and institutional documents addressing training, mentorship, honors, or programs promoting women in STEM. Non-scholarly blog posts and unverifiable sources were excluded.

Contemporary mentee perspective vignette: Included a first-person- narrative from the author describing formative mentoring relationships and institutional recognitions. Only anonymized, non-identifiable personal details were used for analysis; any corroborating records were included only with permission.

Source and recruitment: The contemporary vignette was an author-provided first-person– account from an 18-year-old College student. This narrative was submitted voluntarily to illustrate the mechanisms of mentorship and recognition in a present-day- educational and clinical setting.

Consent and anonymization: The contributor consented to the publication of this first-hand account for the use of the narrative in this study.

Integration with historical data: Themes from the vignette were compared and contrasted with mechanisms identified in the historical review to assess the convergence and divergence between past and contemporary mentorship dynamics.

Limitations: The vignette is a single, non-representative narrative and cannot establish causality. Third, historical sources may reflect selection bias. These limitations inform the cautious interpretation of the conclusions.

## Results/discussion

### Rita Levi-Montalcini: a mentorship legacy

Rita Levi-Montalcini was widely recognized as an inspiring and supportive mentor, particularly noted for her ability to foster scientific growth and personal development in her trainees. She was described as welcoming, warm-hearted, and open to considering others’ ideas without prejudice, encouraging rigorous reasoning and resilience in the face of research challenges. Her approach was characterized by treating young researchers as equals, which contributed significantly to their professional growth and confidence (Malerba, 2022). Levi-Montalcini’s mentorship extended beyond the laboratory (Aloe, 2004); she was deeply committed to education, gender equality, and the empowerment of young scientists and women, exemplified by her establishment of the Rita Levi-Montalcini Foundation for the education of African women (Bentivoglio, 2013). Her mentorship style was marked by factual optimism and a focus on both the starting point and the objective, helping mentees navigate research difficulties (Costa, 2016).

**FIGURE 1:**
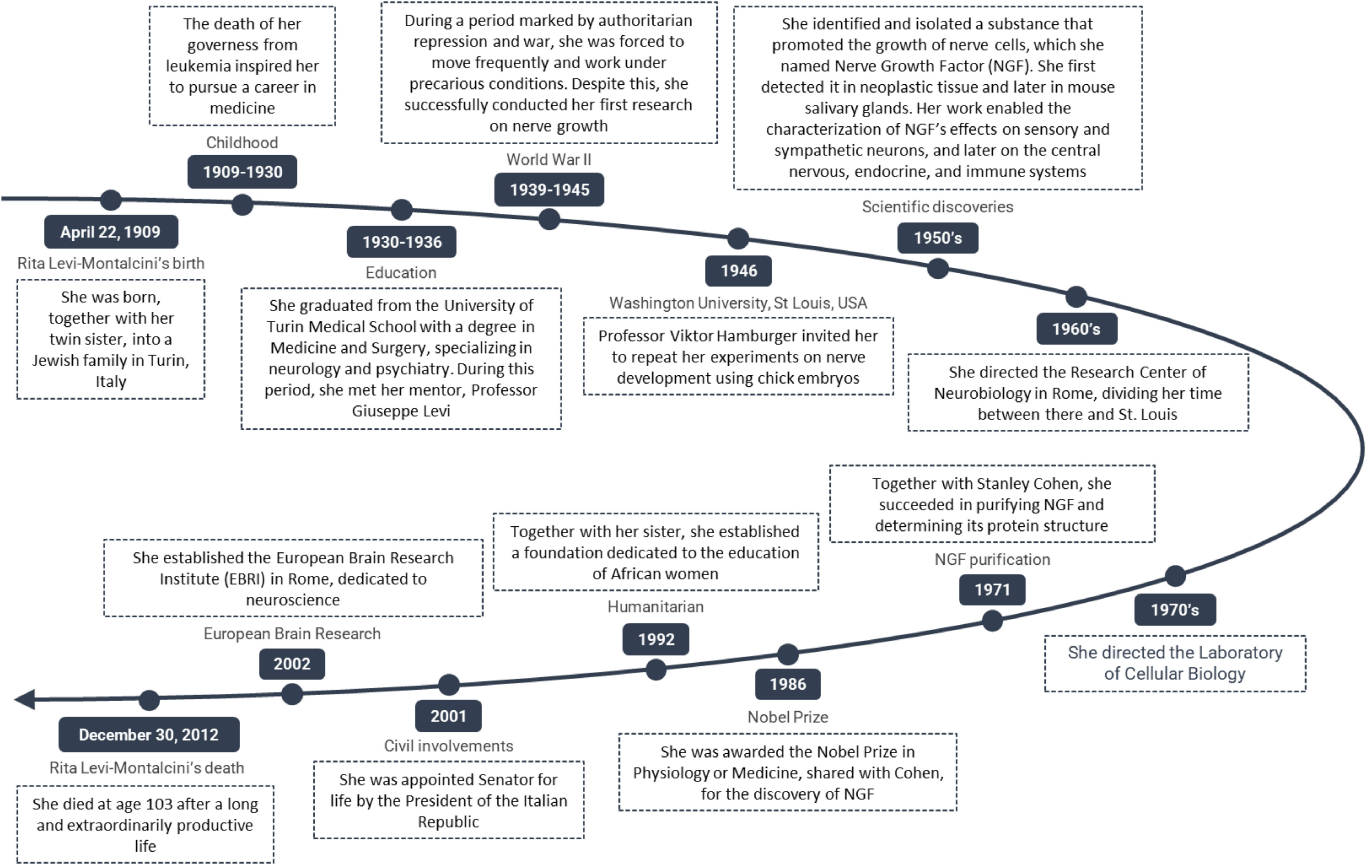
Chronological timeline of Rita Levi-Montalcini’s life, scientific achievements, and legacy.

Her legacy as a mentor is marked by her enduring faith in the potential of young individuals and her advocacy for education as the key to societal development (Bentivoglio, 2013). Long-term collaborators also attest to her scientific and human influence over decades of joint research, highlighting her role in shaping her colleagues’ careers and advancing neuroscience (Aloe, 2021). Table 1 lists key points of Rita Levi Montalcini’s mentoring qualities. A chronological overview of Levi-Montalcini’s- life and career is presented in Figure 1.

**TABLE 1:** Key points of Rita Levi-Montalcini’s mentoring qualities.

Quality/explanation
Mentorship grounded in intellectual equality: treated young researchers as peers, encouraging independent thinking and confidence
Scientific rigor with human warmth: balanced high methodological standards with encouragement and openness to new ideas.
Resilience as a core lesson: transmitted perseverance and courage drawn from her own experience of adversity and exile.
Commitment beyond the laboratory: actively promoted education, gender equity, and the empowerment of young women in science.
Enduring personal influence: shaped not only scientific careers but also values, identity, and long-term engagement in research.

### The foundational influence of Giuseppe Levi

She encouraged open-mindedness, critical reasoning, and resilience in the face of scientific challenges, fostering an environment in which ideas could be freely exchanged and hypotheses considered without prejudice. She had Giuseppe Levi (1872–1965) as her mentor. He played a decisive role in shaping her scientific future. He trained her in histological techniques and instilled in her the rigor and resilience that sustained her during clandestine research under fascist regime.

### Broader impact: Levi’s Nobel laureate mentees

Giuseppe Levi’s mentorship, demonstrates the profound impact a dedicated mentor can have on the development of future Nobel laureates (Aloe, 2021; Chirchiglia et al., 2020; Costa, 2016). Giuseppe Levi played a pivotal role as a mentor, particularly through his training in histological techniques and his emphasis on scientific rigor and resilience, which profoundly shaped the careers of Rita Levi-Montalcini, Salvador Luria, and Renato Dulbecco, all of whom later won Nobel Prizes in medicine.

Levi’s laboratory at the University of Turin was a crucible for scientific talent, where he taught advanced methods for culturing neural tissue and instilled a culture of meticulous observation and experimental discipline in his students. The resilience and scientific rigor that Levi instilled in his students proved particularly crucial during the fascist regime of the late 1930’s. When the 1938 Racial Laws forced Jewish scientists from Italian universities, Levi-Montalcini continued her research clandestinely, applying the techniques and mindset that Levi had taught her (Troiani and Manni, 2007). Costa noted that Levi was the founder of methods for culturing neural tissue and that Levi-Montalcini, Luria, and Dulbecco all trained under him before their Nobel-winning work in the United States (Costa, 2016). Levi’s insistence on technical precision and perseverance in research provided his students with the foundational skills and mindset necessary for ground breaking discoveries. For Rita Levi-Montalcini, his mentorship was critical as she overcame significant personal and professional obstacles, including wartime persecution, to achieve scientific breakthroughs (Chirchiglia et al., 2020) and learn the histological and cell culture techniques that led to the discovery of the nerve growth factor (NGF), a milestone in neurobiology (Aloe, 2021). Levi’s influence extended similarly to Luria and Dulbecco, who both credited their early training in Levi’s lab with their later achievements in molecular biology and virology. Giuseppe Levi’s legacy extends beyond individual mentorship, demonstrating how a dedicated teacher’s technical training and cultivation of scientific character can catalyze multiple generations of discoveries across diverse fields of biomedical research.

Luria earned the 1969 and Dulbecco the 1975 Nobel Prize in Physiology or Medicine (Salvador, 2025; The Nobel Prize, 2025a). Levi-Montalcini was awarded the Nobel Prize in 1986. She was the fourth woman to win the Nobel Prize in Physiology or Medicine. Before her, Gerty Cori won the 1947 Prize, Rosalyn Yalow the 1977 and Barbara McClintock the 1983 Prize (The Nobel Prize, 2025b).

Levi-Montalcini’s commitment to education and advocacy extended beyond her laboratory. She had enduring faith in the potential of young individuals and women, emphasizing education as the key to development and gender equality. She established the Rita Levi-Montalcini Foundation to support the education of African women, further demonstrating her dedication to mentoring and empowering future generations (Bentivoglio, 2013).

Colleagues who worked with her for decades described their experiences as both scientifically and personally enriching, highlighting her ability to inspire and guide researchers throughout their long careers (Aloe, 2021). Her legacy as a mentor is reflected in the continued inspiration she provides to scientists, especially women in neuroscience, and in her lasting impact on the global scientific community (Aloe, 2021; Bentivoglio, 2013; Malerba, 2022). Her mentoring serves as an exemplary model wherein excellence, resilience, and humanism are integrally linked. The principal attributes of this mentoring model are delineated in Figure 1.

### Importance of mentorship for women in STEM (Science, Technology, Engineering, and Mathematics)

Mentorship and recognition are foundational for inclusive excellence in STEM because they empower women to overcome systemic barriers, develop a professional identity, and achieve career success, ultimately enriching scientific innovation and societal progress. Mentorship and recognition are critical for fostering inclusive excellence in STEM, especially for women, because they directly address barriers to belonging, persistence, and advancement that women disproportionately encounter.

Mentorship provides women with access to supportive networks, professional development, and role models, which are essential for building scientific identity, self-efficacy, and motivation to persist in STEM fields (Dennehy and Dasgupta, 2017; Hernandez et al., 2017; Stout et al., 2011). Exposure to female mentors and peers increases women’s sense of belonging and retention, particularly during key transition points, such as early college and graduate training (Dennehy and Dasgupta, 2017; Stout et al., 2011). Long-term studies show that mentoring interventions, even those delivered online during secondary education, have lasting positive effects on women’s choices to pursue STEM majors and careers (Stoeger et al., 2023).

Recognition—through awards, leadership opportunities, and visible authority—helps counteract stereotypes and the lack of legitimacy that women often experience in male-dominated environments (Amon, 2017; Casad et al., 2021; Charlesworth and Banaji, 2019). When women’s contributions are acknowledged, it buffers against the negative effects of stereotype threat and fosters resilience, professional satisfaction, and retention (Amon, 2017). Conversely, a lack of recognition and social capital can limit career advancement and reinforce exclusion (Casad et al., 2021; Charlesworth and Banaji, 2019).

### Scientific proof that mentorship has an influence on the career

There are methodological frameworks that quantify the scientific output, citation impact, and career trajectories of mentees compared to their peers. Ma et al. (2020) developed models explaining 34%–44% of the variance in protégé success across biomedicine, chemistry, math, and physics, demonstrating that mentorship by future prize-winning scientists is associated with a 2×-to-4× increase in protégés’ likelihood of prize-winning, National Academy of Science induction, or achieving superstardom. Chariker et al. (2017) used network analysis of Nobel laureate mentor-mentee relationships to show that Nobel laureates had significantly more Nobel laureate ancestors, descendants, and mentees, with successful mentoring communities identifiable through academic genealogy networks.

Personal mentorship influences student development by providing individualized instrumental and psychosocial support, facilitating professional identity formation, and enhancing academic self-efficacy, and motivation. High-quality mentor-mentee relationships increase the sense of belonging, confidence, and career satisfaction, and improve research productivity and academic performance (Sambunjak et al., 2006). Mentorship operates through role modeling, guided reflection, and scaffolding within practice communities, supporting cognitive and social development (Toh et al., 2022).

### The power of symbolic recognition

Symbolic recognition plays a crucial role in boosting women’s participation and persistence in STEM fields. Awards, honorary degrees, and leadership appointments act as institutional markers of legitimacy and inclusivity. Such recognition helps counteract stereotype threats and invisibility faced by women in male-dominated disciplines, fostering resilience and professional fulfillment. Research shows that acknowledging women’s contributions reduces bias and enhances retention, especially during career transitions. Symbolic recognition serves as both a remedy for systemic inequities and a catalyst for inclusive excellence, transforming private perseverance into public authority. Rita Levi-Montalcini received McGill University’s first honorary doctorate awarded outside Canada in its 190-year history, highlighting her global influence in medicine and women’s advancement in science (McGill, 2011). Recognition signals institutional value of student contributions, increasing motivation and beneficial behaviors (Chaudhry et al., 2024; Jiang et al., 2024). Institutional support fosters a psychological sense of community, mediating student thriving and moderating growth mindset effects (Chaudhry et al., 2024; Jiang et al., 2024). Policy implications include prioritizing structured mentorship programs with protected time and transparent recognition systems (Choi et al., 2019). Institutions should address barriers and ensure inclusivity for diverse students, enhancing professional development and workforce stability (Choi et al., 2019).

## Contemporary mentee perspective

As a contemporary illustration of how mentorship and institutional recognition shape scientific identity and career trajectories, the following first-person- account complements the historical analysis of Rita Levi-Montalcini’s life.

This contemporary vignette mirrors the twofold mechanism identified in Rita Levi-Montalcini’s- trajectory: mentors who teach technical skills and model resilience and institutions that translate private perseverance into public authority through honors and roles. Where Levi Montalcini’s- mentorship and later symbolic recognition created role models and institutional pathways for women in science, the student’s experience demonstrates how modern mentorship and recognition can correct exclusion, build confidence, and create tangible career scaffolding, especially for minorities.

The narrator is an 18-year-old Cégep student born in Canada to a family of Chinese immigrants who encountered early challenges related to belonging, language-related isolation, and recurrent negative feedback regarding academic expression. Two pivotal mentoring relationships—one with a high school French teacher and another with a practicing oncologist—significantly influenced the student’s sense of potential, academic interests, and professional trajectory. Furthermore, encounters with highly esteemed and internationally recognized physicians further shaped her perspective.

### Early barrier and turning point

Language difficulties and repeated criticism had made this student’s writing a source of shame rather than a tool for expression. The student’s Secondary 2 French teacher changed this dynamic by treating writing as a vehicle for meaning rather than merely a set of rules. The teacher’s specific praise for her served as a catalytic moment: validation shifted the student’s relationship to language from avoidance to curiosity and opened a new avenue for intellectual engagement.

### Experiential mentorship in medicine

The second pivotal relationship began when the student reached out to a local oncologist and was invited to shadow the oncologist in the oncology department. The clinician translated complex procedures into accessible explanations, demonstrated clinical communication with patients, and offered ongoing career guidance and practical support for the applications. This hands-on- exposure demystified oncology, provided concrete skills and vocabulary, and created a sense of belonging in a field that had previously felt remote and intimidating.

Another influential figure was Dr. Sue Yom, who held both an MD and a Ph.D. in English. Dr. Yom is a distinguished American radiation oncologist and academic leader at the University of California, San Francisco, renowned for her clinical expertise in head and neck cancers and thoracic cancers. She occupies national trial-leadership positions and serves as the Editor-in-Chief of one of the two major peer-reviewed journals in her field. In this context, Dr. Yom serves as a public and interdisciplinary role model whose career seamlessly integrates science and communication. Her example, which combines clinical oncology with a strong focus on clear writing and public engagement, affirmed the student’s dual interests in literature and medicine. Observing a prominent oncologist who publishes, speaks, and teaches with clarity enabled the student to envision a professional identity that does not necessitate choosing between the humanities and the sciences. Dr. Yom’s visibility and approachability underscored the notion that effective patient care requires both technical expertise and the ability to convey complex information with compassion and precision. As a role model, she provided a framework for incorporating communication skills into clinical practice and utilizing public platforms to influence professional norms.

Mechanisms of influence: The two personal mentors employed complementary mechanisms to facilitate their development. The teacher offered psychological scaffolding, which included encouragement, reframing, and identity work, thereby enhancing self-efficacy and promoting intellectual risk taking. Conversely, the clinician provided sponsorship and access, offering opportunities to observe professional practice, facilitating introductions to conferences and role models, and providing concrete advice on curriculum vitae and applications. Collectively, the combination of encouragement and sponsorship transformed initial interest into actionable steps and measurable results.

### Contemporary relevance of Rita Levi-Montalcini’s mentorship model

Rita Levi-Montalcini’s mentoring style, characterized by intellectual equality, warmth, and resilience, provides a framework for today’s scientific community. Her clear communication and advocacy for education serves as an example for trainees lacking direct role models. For busy principal investigators, her approach shows that effective mentorship requires intentionality: treating trainees as partners, providing meaningful feedback, and demonstrating curiosity. Her journey from bedroom experiments to Nobel Prize resonates with undergraduate students, showing that scientific discovery is accessible through persistence and creativity. For immigrant women balancing multiple responsibilities, Levi-Montalcini’s life demonstrates possibility despite systemic barriers. Her support for women’s education, including her foundation for African women, emphasizes that scientific potential transcends origin and circumstance.
